# Whole Genome Expression Array Profiling Highlights Differences in Mucosal Defense Genes in Barrett's Esophagus and Esophageal Adenocarcinoma

**DOI:** 10.1371/journal.pone.0022513

**Published:** 2011-07-28

**Authors:** Derek J. Nancarrow, Andrew D. Clouston, B. Mark Smithers, David C. Gotley, Paul A. Drew, David I. Watson, Sonika Tyagi, Nicholas K. Hayward, David C. Whiteman, for the Australian Cancer Study and the Study of Digestive Health

**Affiliations:** 1 Oncogenomics, Queensland Institute of Medical Research, Herston, Queensland, Australia; 2 School of Medicine, Southern Clinical Division, Princess Alexandra Hospital, Woolloongabba, Queensland, Australia; 3 Upper Gastrointestinal and Soft Tissue Unit, Princess Alexandra Hospital, and Department of Surgery, University of Queensland, Princess Alexandra Hospital, Woolloongabba, Queensland, Australia; 4 Department of Surgery, University of Adelaide, Royal Adelaide Hospital, Adelaide, South Australia, Australia; 5 School of Nursing and Midwifery, Flinders University, Bedford Park, South Australia, Australia; 6 Department of Surgery, Flinders University, Bedford Park, South Australia, Australia; 7 Cancer and Population Studies, Queensland Institute of Medical Research, Herston, Queensland, Australia; Technische Universität München, Germany

## Abstract

Esophageal adenocarcinoma (EAC) has become a major concern in Western countries due to rapid rises in incidence coupled with very poor survival rates. One of the key risk factors for the development of this cancer is the presence of Barrett's esophagus (BE), which is believed to form in response to repeated gastro-esophageal reflux. In this study we performed comparative, genome-wide expression profiling (using Illumina whole-genome Beadarrays) on total RNA extracted from esophageal biopsy tissues from individuals with EAC, BE (in the absence of EAC) and those with normal squamous epithelium. We combined these data with publically accessible raw data from three similar studies to investigate key gene and ontology differences between these three tissue states. The results support the deduction that BE is a tissue with enhanced glycoprotein synthesis machinery (DPP4, ATP2A3, AGR2) designed to provide strong mucosal defenses aimed at resisting gastro-esophageal reflux. EAC exhibits the enhanced extracellular matrix remodeling (collagens, IGFBP7, PLAU) effects expected in an aggressive form of cancer, as well as evidence of reduced expression of genes associated with mucosal (MUC6, CA2, TFF1) and xenobiotic (AKR1C2, AKR1B10) defenses. When our results are compared to previous whole-genome expression profiling studies keratin, mucin, annexin and trefoil factor gene groups are the most frequently represented differentially expressed gene families. Eleven genes identified here are also represented in at least 3 other profiling studies. We used these genes to discriminate between squamous epithelium, BE and EAC within the two largest cohorts using a support vector machine leave one out cross validation (LOOCV) analysis. While this method was satisfactory for discriminating squamous epithelium and BE, it demonstrates the need for more detailed investigations into profiling changes between BE and EAC.

## Introduction

Over recent decades the incidence of esophageal adenocarcinoma (EAC) has increased rapidly in western societies [1], [2], [3], but whilst recent evidence suggests that the rate may have stabilized [4], [5] this cancer now represents a significant health burden. Epidemiological data relate the increased prevalence to factors such as smoking, obesity and gastro-esophageal reflux [6], [7], [8], [9].

The biology leading to EAC development is not fully understood (reviewed in Reid et al., 2010 [10] & Phillips et al., 2010 [11]). What is known presents a multistep process which begins when the normal squamous epithelium of the esophagus is repeatedly damaged by gastro-esophageal reflux. In a subset of individuals the damaged epithelium then undergoes a process of metaplasia with replacement by Barrett's esophagus (BE), a columnar epithelial tissue with intestinal metaplasia. In a subset of cases BE undergoes a malignant progression resulting in the formation of EAC (estimated to occur in 0.5–2.0% of patients with BE per year). This transformation can be histologically observed as progressive dysplasia within the columnar phenotype. While the general histopathological evolution from BE through high grade dysplasia to EAC is well described, the underlying biological mechanisms remain elusive, but suggest considerable variation in relation to expression of specific gene products and the disease stage at which they are important. Furthermore, while the presence of BE does confer a substantially (perhaps 30–40 fold) higher risk of developing EAC [12], the majority of subjects with BE die from other causes (reviewed in Reid 2010 [10]).

The use of genome-wide gene expression arrays, in conjunction with bioinformatics, has allowed groups of genes to be collectively associated with the initiation of several common cancer types. Comparing gene expression profiles between the key histological stages in the progression towards EAC is one way to infer the biological processes involved, as well as affording the opportunity to identify potential therapeutic targets for development on novel treatments for EAC. Several research groups have attempted this [13], [14], [15], [16], [17], [18], [19], [20], but identifying the key genetic factors has been hampered by the relatively limited overlap between the gene lists from the various profiling studies [21]. While exhibiting different experimental designs, the studies have generally focused on distinguishing squamous mucosa from BE, and from EAC; the accepted histologic tissue stages. We hypothesized that applying a standardized approach to the analysis of data from multiple studies would be more likely to produce a robust core gene list which differentiates the three tissue stages under investigation. Here we analyze gene expression data from our sample of patients sourced from a number of centers in Australia, and compare it to several similar datasets that have been released into the public domain [13], [16], [18]. The aim of this study was to use the combined expression profiling data to identify a concordant set of ontology based gene clusters which distinguish between the key histological tissue types (squamous, BE and EAC), as well as highlighting some individual gene differences, across the reported studies.

## Methods

### Participants

The biopsies used to generate our gene expression data were collected from a subset of participants in the Study of Digestive Health (SDH), methods for which have previously been described in detail [6], [7]. Approval for this study was obtained from the research ethics committees of the Queensland Institute of Medical Research (Queensland Institute of Medical Research Human Research Ethics Committee), Flinders University (Flinders Clinical Research Ethics Committee) and participating hospitals; Princess Alexandra Hospital (Metro South Health Service District Human Research Ethics Committee), Mater Private Hospital (Mater Health Services Human Research Ethics Committee), Royal Adelaide Hospital (Royal Adelaide Hospital Research Ethics Committee), Flinders Medical Centre (Flinders Clinical Research Ethics Committee) and The Repatriation General Hospital (currently managed by a caretaker committee; Flinders Clinical Research Ethics Committee). Prior to undergoing upper gastrointestinal endoscopy, participants gave written informed consent for additional biopsies for this study to be taken during their medical procedure. Patients eligible for inclusion were those aged 18 to 80 years with a diagnosis of histologically confirmed BE (specialized intestinal metaplasia and negative for dysplasia n = 22) or EAC (n = 23). Control squamous tissues (S) were obtained from patients who had similarly undergone upper gastrointestinal endoscopy but in whom no abnormalities were detected by either endoscopic or histopathologic examination (n = 9). The patients in the three study groups; squamous tissue controls (S), BE without dysplasia (BE) and EAC, demonstrated gradients for both age (51, 61, and 68 years for mean group ages respectively) and gender ratio (56%, 68% and 96% male predominance, respectively) consistent with epidemiology studies [22], [23].

### Study of Digestive Health biopsy samples

The SDH sample comprises 54 biopsy specimens, collected from 54 individuals (this sample set is referred to as SDH-54). The location of the collection site (distance in cm from incisors and distance from gastro-esophageal junction) and macroscopic appearance of the tissue (squamous, columnar or EAC) were reported for each biopsy by the endoscopist on a standardized form. Biopsies were placed in RNAlater (Ambion, Austin, TX) immediately upon collection and left at 4°C overnight. Samples were then stored at −20°C before removal of excess RNAlater and long-term storage at −70°C.

All 23 EAC biopsies used in this study were collected prior to the initiation of neoadjuvant therapy. The histopathology for most participants (48 of 54) was reviewed by a single experienced pathologist (A.D.C.) using H&E slides derived from separate biopsies taken at the same time and from the same esophageal level as the research biopsy. For the remainder of tissues, pathology review was based on surgical resection specimens (6 of 54). Biopsies from the patient controls were reviewed to confirm that there was no evidence of either esophagitis or BE. BE biopsies were reviewed to exclude patients with dysplasia. The past medical history of patients in a surveillance program was reviewed. All 22 BE participants in the SDH-54 had no prior history of dysplasia and all histologically assessed BE biopsies were confirmed to be negative for dysplasia. For each EAC biopsy we established that the tumor content was more than 50%, based on assessment of DNA copy number data derived from the same biopsy using the procedure outlined previously [24].

### RNA isolation

Whole esophageal biopsies were disrupted using a mechanized tissue fractionator (Qiagen, Germany) in a 1.5 ml microfuge tube with a single 5 mm stainless steel ball-bearing according to the manufacturer's protocol. Nucleic acid (both genomic DNA and total RNA) was extracted using AllPrep (Qiagen) columns and procedures as per the manufacturer's instructions. Samples yielding 1 ug or more of total RNA were used for expression profiling.

### Bead array hybridization

The Sentrix Human-6 Expression BeadChip system, version 1 (Illumina Inc, San Diego, CA) was used, as per the protocol set out in Gene Expression Omnibus (GEO) platform ID numbers: GPL2507 and GPL6097. Briefly, 90 ng of total RNA were applied to the Illumina RNA Amplification Kit (Ambion Inc, Austin, TX) supplied with the Beadchips, to perform double-stranded cDNA generation, followed by *in vitro* transcription to synthesize cRNA, as per the manufacturer's instructions. The size and integrity of the cRNA was assessed by liquid chromatography using a Bioanalyzer (Agilent Technologies, Santa Clara, CA) as described in the TotalPrep RNA Amplification Kit booklet (Illumina catalog; #IL1791). All samples considered for microarray hybridization showed the expected profile with the majority of fragments in the range of 1000–1500 nt.

The purified cRNA was then labeled and hybrized to the Beadchips for 17 hours at 42°C in a rotating oven (Thermo Fisher Scientific, Waltham, MA). Chips were then washed, stained, and scanned according to the protocol described in the Whole Genome Gene Expression for BeadStation Manual, Revision D (Illumina).

GenomeStudio software, version 2.0 (Illumina) was used to extract raw signal intensity data. Quality control plots within GenomeStudio showed acceptable signal strengths for all 54 samples. Barbosa-Morais and co-workers [25] have demonstrated that a large number of probes on the Human-6 version 1 chips do not bind uniquely to the transcriptome. We have chosen to include only those probes deemed to be ‘perfect’ with regards to these analyses (n = 25049); those that bind uniquely and have a perfect match to the consensus genome [25]. Both raw and processed expression data for the SDH-54 cohort are available in GEO series GSE28302.

### Preparation of comparison cohorts

**Figure 1:**
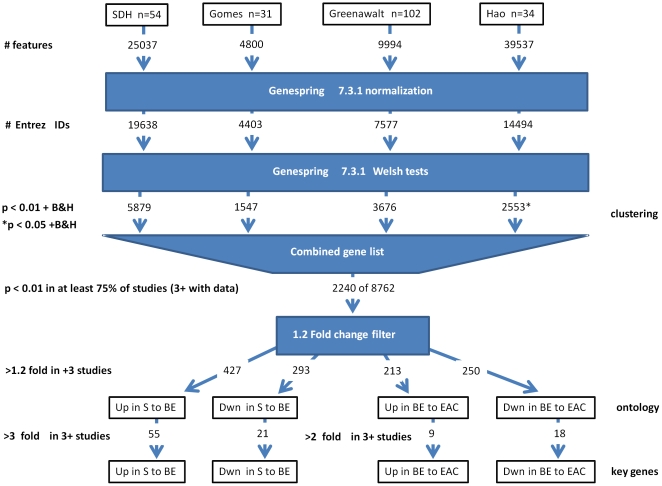
Study schema to combine 4 EAC expression profiling studies. mRNA profiling data for squamous, BE and EAC samples from the new cohort (SDH-54) and three similarly size or larger samples for which raw data were available (Gomes -34, Greenawalt-102 and Hao-41). In each case profiling data were analyzed using standard ANOVA methodologies to generate gene lists that discriminated the three tissue types in each cohort (Figure 2). Gene lists were then overlapped and the most frequently discriminating genes, those with >1.2 fold tissue group differences in at least 3 cohorts (Table S1), were used for ontology studies. More stringent fold-change thresholds were used to isolate the peak genes that discriminate squamous from BE (Table 1 & 2) and BE from EAC (Table 3) tissue groups. * The Hao-34 sample set required a less stringent (p<0.05) threshold in order to generate genes.

We restricted primary analysis to published cohorts with publically available raw data employing genome-wide expression array platforms on individual, histologically verified normal esophageal squamous, BE and EAC tissues. To ensure adequate power to detect discriminatory gene profiles we further restricted inclusion to those cohorts with at least 250 genes passing the B&H false discovery rate adjusted threshold of p<0.05 for a 3 group (squamous, BE and EAC) Welsh test comparison. We identified 3 studies which met these criteria [13], [16], [18]. Several of these studies analyzed additional tissue types (e.g. gastric or intestinal or squamous cell carcinoma biopsy samples) which we excluded to allow a consistent comparison between normal esophageal squamous, BE and EAC tissues. To indicate that we were comparing to a subset of the originally published work we refer to each study by first author surname, followed by total number of squamous, BE and EAC samples (Gomes-41, Hao-34 & Greenawalt-102). We compared these studies to the 54 individual samples outlined above (SDH-54), making a combined total of 72 squamous, 81 BE and 78 EAC tissue samples. Since numerous procedural differences existed between each of the studies (including sample selection, sample preparation, array platforms, and bioinformatic annotation methods), it was not possible to conduct a direct comparison of samples. Thus we analyzed each cohort separately and collated the resulting independent gene lists into a single master list, as illustrated in Figure 1. In each instance, we used the annotation data from each study, combined with DAVID [26], [27] and/or ACID [28] bioinformatics databases to link chip probe IDs or accession numbers to active Entrez gene IDs. In this way we were able to harmonize studies across very different chip technologies into unified gene lists. Probes which could not be linked to an Entrez ID, or that associated with multiple IDs were excluded from the final tabulated lists for each study (Figure 1).

### Data preprocessing

Figure 1 summarizes our analytic approach to identifying the most frequently involved genes and pathways in the progression to EAC. Our goal was to apply a standard set of expression profiling adjustments and gene-selection criteria to each of the 4 cohorts in order to gain a comparable gene list from each study. Pre-background adjusted, tab-delineated data for each of the 4 cohorts was imported into GeneSpring GX version 7.3.1 (Agilent Technologies, Inc., CA, USA) and normalized (logarithm to the base 2). Signals were corrected for background (<0.01 adjusted to 0.01) and normalized for intensity (Lowess residual to the 50^th^ percentile) within GeneSpring.

### Supervised sample clustering across 4 cohorts

We generated a visual comparison of sample relationships within each cohort, using a consistent gene selection approach, to study misclassification of individual samples. Given that the number of Entrez gene IDs within the 4 genome-wide studies varied from ∼4.4 K to 19.6 K, we chose to use the Welsh test (ANOVA assuming unequal variance), with a Benjamini & Hochberg (B&H) false discovery rate (FDR) adjustment [29], to identify genes that significantly discriminated between the three tissue states (squamous, BE & EAC) in each study. A B&H adjusted p value threshold of p<0.01, was used for each cohort, with the exception of the Hao-34 cohort, which required a B&H filter of p<0.05 to generate a gene list. We then used a Tukey post hoc analysis to determine the mean expression values for each sample group.

Genespring ‘standard’ clustering (a variant Pearson algorithm) was applied to the B&H filtered discriminatory gene list from each cohort to generate supervised dendrograms using average linkage. Unsupervised clustering (all chip elements) was also performed for each study, as a comparison.

### Generating a consensus gene list for ontology

Our aim was to identify ontology-based gene clusters with consistent evidence of differential expression levels between squamous and BE, or BE and EAC. We generated a master list of Entrez IDs present in at least three studies (n = 8762). For each of these genes we recorded the number of studies in which it was present, and the number of studies for which it passed the Welsh test threshold. We considered that genes (Entrez IDs) which passed the threshold in 75% of studies provided nominal support for differential expression. This equates to at least 3 of the four cohorts providing evidence of differential expression. There were 2240 Entrez IDs which met these criteria.

For the purpose of tracking gene ontology changes we catalogued genes from our differential expression list with respect to the direction of fold change (>1.2-fold increase/decrease) when comparing squamous to BE, or BE to EAC mean group differences, for each study. We noted each instance where there was a >1.2-fold mean group difference, in the same direction (either increasing or decreasing) in at least 75% of the studies (Table S1). Each of these four lists was then subjected to DAVID ontology analysis, using the default feature listings and algorithm settings, with the whole human genome as background. Ontology categories with FDR adjusted (Benjamini) p values <0.05 were recorded.

### Identifying the most discriminating gene subset

To identify the most consistently altered individual genes we chose the subset with either a >3-fold change in at least 3 of the 4 cohorts for squamous to BE comparisons (Table 1 for those within decreased expression in BE & Table 2 for genes with increased BE expression, relative to squamous), or >2-fold change in 3 or more cohorts for BE to EAC comparisons to demonstrate strong, reproducible expression differences (Table 3). There is no standard fold-change filter applied consistently in the literature: both two-fold and three-fold mean group expression filters are prevalent. Given that the squamous/BE discrimination is one of tissue type, while BE/EAC relates to cancer progression there is no imperative for the thresholds to be the same. We used different fold-change thresholds for the two comparisons to restrict gene list lengths, given that there were much stronger associations when contrasting squamous and BE. We annotated this subset of genes to determine the relevant ontology groups using the methodologies described above.

**Table 1:** Peak Genes Decreased (Fold change ratio less than -3 in at least 3 studies) for Squamous verse BE Group Comparisons across 4 Cohorts.

			ANOVA p value S vs BE vs EAC#					Mean Fold Change Ratio BE/S# ∧				Independent
Entrez ID	SYMBOL	Fold in BE	SDH	GOMES	GREENAWALT	HAO*	p<0.01 Count*	SDH	GOMES	GREENAWALT	HAO	profiling references
360	AQP3	down	1.3E-10	0.0004	2.9E-07	0.009	4/4	−7.6	−3.4	−6.6	−2.3	[15]
379	ARL4D	down	7.8E-11		6.8E-14	0.043	3/4	−3.6		−7.1	−10.7	[15], [17]
390	RND3	down	9.4E-05	—	1.5E-06	0.002	3/3	−3.5	—	−4.6	−4.8	
646	BNC1	down	0.0012	6E-05	2.2E-11	0.041	4/4	−1.3	−3.3	−6.1	−4.1	
810	CALML3	down	2.6E-09	—	3.7E-07	0.033	3/3	−22.2	—	−7.2	−3.1	[15]
874	CBR3	down	1.2E-06	0.0001	1E-09	0.028	4/4	−3.0	−4.5	−3.6	−4.1	[17]
978	CDA	down	3.2E-07	2E-05	1E-10		3/4	−5.2	−3.6	−4.2		[17]
1382	CRABP2	down	1.5E-09		2.2E-12	0.024	3/4	−12.5		−6.0	−6.7	[17]
1410	CRYAB	down	8.3E-11	—	0.00037	0.035	3/3	−6.4	—	−3.7	−4.2	
2012	EMP1	down	7.4E-08	9E-05	1.3E-07	—	3/3	−6.8	−33.8	−3.4	—	[15], [17], [36]
2125	EVPL	down	5.3E-10	0.003	7.2E-08	0.027	4/4	−7.0	−7.8	−5.3	−5.5	[30]
5292	PIM1	down	2.8E-06	0.0001	5.9E-05	0.028	4/4	−3.7	−4.5	−8.0	−10.2	[15]
5493	PPL	down	6.9E-10	—	4.4E-09	0.017	3/3	−11.8	—	−5.6	−5.5	[15], [17]
10848	PPP1R13L	down	6.4E-06	0.0018	1.6E-10		3/4	−3.1	−3.7	−4.7		
23136	EPB41L3	down	6.7E-12	—	3.5E-11	0.012	3/3	−5.4	—	−5.5	−9.8	
23328	SASH1	down	1.7E-05	8E-06	5.5E-10	0.01	4/4	−3.2	−7.0	−7.3	−8.2	[15]
23650	TRIM29	down	2.1E-08		2.7E-08	0.019	3/4	−5.5		−7.1	−4.4	[15], [17]
26085	KLK13	down	9.2E-09	2E-05	0.00055		3/4	−10.0	−30.9	−5.7		[15]
26353	HSPB8	down	1.1E-08		2.2E-12	0.02	3/4	−6.0		−7.8	−8.2	
27076	LYPD3	down	5.8E-09	—	1.6E-14	0.002	3/3	−7.8	—	−7.3	−7.9	
57162	PELI1	down	0.0019	0.0029	4.1E-08	0.005	4/4	−2.0	−3.8	−8.1	−3.8	

*For Hao-34 p<0.05 was required for any genes to pass threshold in the presence of B&H FDR.

# “—” represents genes not present on the array in question while blanks represent non-significant genes for a given study.

∧ Extreme fold change values may be the result due to rounding of non-expressed values (>0.01 = 0.01).

**Table 2:** Peak Genes Increased (Fold change ratio greater than 3 in at least 3 studies) for Squamous verse BE Group Comparisons across 4 Cohorts.

			ANOVA p value S vs BE vs EAC#					Mean Fold Change Ratio BE/S# ∧				Independent
Entrez ID	SYMBOL	Fold in BE	SDH	GOMES	GREENAWALT	HAO*	p<0.01 Count*	SDH	GOMES	GREENAWALT	HAO	profiling references
126	ADH1C	up	1.6E-12	—	8.1E-07	0.007	3/3	24.2	—	13.9	4.3	
283	ANG	up	1.8E-13		8.4E-10	0.005	3/4	10.8		3.4	12.3	
489	ATP2A3	up	1.5E-11	—	2.3E-08	0.021	3/3	5.7	—	6.4	7.8	[14], [15]
563	AZGP1P1	up	4.2E-06		3.6E-06	0.022	3/4	3.5		3.7	9.6	
629	CFB	up	6.9E-11	—	7.1E-07	0.016	3/3	6.0	—	3.2	6.1	
760	CA2	up	2.7E-08	—	3.4E-09	0.032	3/3	12.2	—	13.1	11.8	[15], [17], [34], [36]
1510	CTSE	up	1.5E-08	—	6.1E-09	0.006	3/3	3.2	—	29.8	41.5	[15], [19]
1612	DAPK1	up	1.3E-10		2.2E-10	0.002	3/4	4.7		5.0	3.3	
1803	DPP4	up	2.1E-08	0.0004	7.2E-07		3/4	3.6	3.6	19.3		
2203	FBP1	up	1.3E-07		6E-08	0.009	3/4	12.5		8.4	8.3	
2331	FMOD	up	3.8E-09		3.1E-06	0.016	3/4	4.9		9.4	14.7	[36]
2705	GJB1	up	8.3E-13		1.5E-06	0.024	3/4	3.7		12.4	6.6	[17]
3158	HMGCS2	up	5.1E-12	0.0004	4.4E-11	0.048	4/4	14.6	3.3	19.0	21.6	
3171	FOXA3	up	6.6E-13	—	3.9E-09	0.006	3/3	5.6	—	12.9	123.6	[17]
3217	HOXB7	up	1.7E-11	0.0005	9.7E-13	0.037	4/4	3.4	1.3	7.6	9.3	[15]
3373	HYAL1	up	4.4E-11	—	3.6E-08	0.017	3/3	9.0	—	6.9	11.9	[30]
3783	KCNN4	up	4.5E-09	—	1E-09	0.006	3/3	3.1	—	3.7	9.2	
3960	LGALS4	up	3.9E-10		5E-11	0.026	3/4	55.2		32.1	7.0	[19], [20], [35]
4060	LUM	up	6.4E-05	0.0003	5.2E-05		3/4	4.9	3.3	3.2		
4584	MUC3B	up	1.4E-07	—	1.7E-08	0.048	3/3	12.0	—	17.7	11.2	
4588	MUC6	up	1.6E-11	—	6.1E-05	0.043	3/3	43.8	—	26.6	3.6	[21], [30]
4640	MYO1A	up	2.7E-11		5.3E-06	0.037	3/4	11.0		14.6	11.5	
4907	NT5E	up	3.1E-07	7E-05	2.8E-06	0.032	4/4	3.0	4.3	10.4	3.9	
5264	PHYH	up	3.3E-11		2.7E-10	0.036	3/4	3.8		4.2	13.5	
5332	PLCB4	up	4.1E-11	—	1.8E-08	0.005	3/3	3.6	—	4.2	19.7	
5997	RGS2	up	0.0017		1.5E-06	0.019	3/4	3.7		9.8	4.3	
6035	RNASE1	up	8E-10		3.4E-09	0.016	3/4	15.2		6.1	14.6	[36]
6690	SPINK1	up	1.4E-10	—	7.8E-07	0.025	3/3	41.6	—	22.4	26.4	[19], [21]
6819	SULT1C2	up	3E-10		2.4E-09	0.009	3/4	5.2		58.5	28.3	[19]
7031	TFF1	up	1.7E-08	—	2.5E-10	0.005	3/3	123.7	—	53.7	7.4	[19], [20], [21], [34], [35], [36]
7429	VIL1	up		2E-05	1.8E-09	0.033	3/4		5.6	21.2	34.1	[19], [36]
8513	LIPF	up	5.2E-08	—	0.00013	0.045	3/3	46.3	—	209.9	13.1	[36]
8842	PROM1	up	1.1E-11		1.4E-09	0.023	3/4	10.1		18.5	4.8	[19]
8876	VNN1	up	4.6E-08	—	2.1E-07	0.045	3/3	3.4	—	10.8	4.6	[20]
8985	PLOD3	up	7.1E-12	—	7.7E-11	0.018	3/3	3.3	—	3.8	4.3	
8991	SELENBP1	up	1.4E-12	—	3.5E-09	0.002	3/3	6.3	—	5.5	8.4	
10008	KCNE3	up	1E-11	—	6.4E-08	0.027	3/3	9.2	—	8.6	14.4	
10099	TSPAN3	up	8.6E-11		2.1E-06	0.034	3/4	5.8		3.8	10.1	[20]
10103	TSPAN1	up	1.3E-09	—	3.5E-11	0.004	3/3	58.3	—	63.5	54.5	[19], [21], [36]
10396	ATP8A1	up		1E-06	7.7E-06	0.041	3/4		24.6	3.5	6.8	
10551	AGR2	up	5.6E-14	—	1.4E-11	0.003	3/3	35.5	—	27.4	17.0	[17]
10723	SLC12A7	up	6.1E-10	—	9E-11	0.007	3/3	3.6	—	4.1	4.1	
10788	IQGAP2	up	2.3E-11	—	8.9E-12	0.027	3/3	5.0	—	15.6	4.5	[19]
10954	PDIA5	up	2.2E-13	—	2.3E-08	0.026	3/3	4.0	—	4.8	7.8	
11015	KDELR3	up	6.2E-13		9.9E-11	0.025	3/4	6.0		8.0	9.4	[15]
11145	PLA2G16	up	6.2E-13	—	1.3E-10	0.009	3/3	7.3	—	8.4	7.6	
11199	ANXA10	up	1.4E-11	—	3.5E-09	0.009	3/3	26.7	—	19.4	14.8	[15], [19], [35], [36]
25945	PVRL3	up	9.9E-12	—	4.3E-07	0.009	3/3	3.6	—	8.8	47.9	
30011	SH3KBP1	up	1.5E-05		2.4E-11	0.009	3/4	3.6		3.8	5.7	
51703	ACSL5	up	1.2E-11	0.0004	4.3E-07	0.037	4/4	7.6	4.2	4.9	8.3	
54474	KRT20	up	1.9E-10		3.5E-07	0.015	3/4	13.9		58.9	25.2	[14], [19], [34], [35]
56654	NPDC1	up	4.2E-11	—	2.2E-05	0.016	3/3	3.9	—	3.8	4.0	
81618	ITM2C	up	4.2E-13	—	1.3E-08	0.018	3/3	7.4	—	5.2	4.0	
118429	ANTXR2	up	8.3E-06	—	9.9E-09	0.019	3/3	3.2	—	3.5	7.8	
445329	SULT1A3	up	1.2E-07	—	4.9E-05	0.029	3/3	3.5	—	3.8	5.0	

*For Hao-34 p<0.05 was required for any genes to pass threshold in the presence of B&H FDR.

# “—” represents genes not present on the array in question while blanks represent non-significant genes for a given study.

∧ Extreme fold change values may be the result due to rounding of non-expressed values (>0.01 = 0.01).

**Table 3:** Peak Genes (Fold change ratio greater than 2 in at least 3 studies) for BE verse EAC Group Comparisons across 4 Cohorts.

			ANOVA p value S vs BE vs EAC#					Mean Fold Change Ratio EAC/BE# ∧				Independent
Entrez ID	SYMBOL	Fold in BE	SDH	GOMES	GREENAWALT	HAO	p<0.01 Count*	SDH	GOMES	GREENAWALT	HAO	profiling references
125	ADH1B	down	3E-05		0.002	0.0119	3/4	−2.3		−2.1	−3.6	
126	ADH1C	down	2E-12	—	8E-07	0.0072	3/3	−4.8	—	−2.1	−6.7	
760	CA2	down	3E-08	—	3E-09	0.0323	3/3	−5.1	—	−3.6	−4.3	[15], [17], [34], [36]
957	ENTPD5	down	2E-07	—	3E-06	0.0118	3/3	−2.2	—	−3.5	−22.5	
1159	CKMT1A	down	0.0022	0.0064	2E-06	0.007	4/4	−1.9	−2.1	−2.0	−2.3	
1646	AKR1C2	down	0.002		0.0047	0.0219	3/4	−4.0		−2.1	−2.7	
3248	HPGD	down	7E-06	0.0008	2E-08		3/4	−5.3	−7.8	−6.1		
3373	HYAL1	down	4E-11	—	4E-08	0.0165	3/3	−2.9	—	−3.8	−7.2	[30]
4588	MUC6	down	2E-11	—	6E-05	0.043	3/3	−13.1	—	−4.2	−11.6	[21], [30]
4640	MYO1A	down	3E-11		5E-06	0.0365	3/4	−2.3		−2.9	−5.2	
5873	RAB27A	down	3E-08		3E-05	0.005	3/4	−2.3		−2.2	−2.5	
6819	SULT1C2	down	3E-10		2E-09	0.0086	3/4	−2.2		−2.8	−13.3	[19]
7031	TFF1	down	2E-08	—	3E-10	0.0049	3/3	−5.7	—	−3.2	−5.1	[19], [20], [21], [34], [35], [36]
8513	LIPF	down	5E-08	—	0.0001	0.0447	3/3	−32.2	—	−5.2	−154.0	[36]
11199	ANXA10	down	1E-11	—	4E-09	0.0089	3/3	−6.5	—	−3.1	−11.1	[15], [19], [35], [36]
23584	VSIG2	down	3E-09		1E-08	0.012	3/4	−6.0		−2.3	−3.5	
54474	KRT20	down	2E-10		4E-07	0.0145	3/4	−4.2		−7.5	−2.8	[14], [34], [35], [36]
57016	AKR1B10	down	1E-06		0.0003	0.018	3/4	−5.7		−2.4	−4.3	
1278	COL1A2	up	0.0007	—	4E-07	0.0287	3/3	2.8	—	3.8	2.2	
1282	COL4A1	up	7E-05	7E-06	0.0078	0.046	4/4	4.8	3.2	3.2	10.3	
1284	COL4A2	up	0.0003	—	3E-07	0.0105	3/3	2.1	—	2.4	5.2	[17]
1290	COL5A2	up	0.0007	—	0.0074	0.0266	3/3	2.4	—	17.5	5.8	
1293	COL6A3	up	0.0012		1E-06	0.0396	3/4	2.0		2.1	7.3	
3490	IGFBP7	up	0.0001	—	3E-08	0.0366	3/3	2.5	—	2.2	5.0	[17], [36]
5328	PLAU	up	2E-05	0.0047	0.0004		3/4	4.1	3.5	4.9		
6772	STAT1	up	0.0005	—	2E-05	0.0442	3/3	2.1	—	3.7	3.7	
23636	NUP62	up	2E-05		3E-05	0.0485	3/4	2.6		4.0	2.4	

*For Hao-34 p<0.05 was required for any genes to pass threshold in the presence of B&H FDR.

# “—” represents genes not present on the array in question while blanks represent non-significant genes for a given study.

∧ Extreme fold change values may be the result due to rounding of non-expressed values (>0.01 = 0.01).

### Literature comparison

In order to compare our peak genes to those of previous reports, we identified 11 reports based on whole genome expression arrays [14], [15], [17], [19], [20], [21], [30], [31], [32], [33], [34] independent of those for which we have included samples in the current study [13], [16], [18], and 2 reports based on Serial Analysis of Gene Expression (SAGE) of whole-genome profiling studies [35], [36] involving EAC and/or BE. We have scanned these reports for mention of official HUGO Gene Nomenclature Committee (HGNC) [37] human gene symbols or names downloaded from http://www.genenames.org in December 2010. In each case we excluded text matches arising within methods or supplementary data in order to focus on those genes the authors of each manuscript deemed worthy of mention (including Figures).

Gene text searches were conducted in two stages, an initial automated screening, followed by manual confirmation of genes present in at least three studies. We used version 7.1 of the Spell Checker Oriented Word Lists (SCOWL) library (http://wordlist.sourceforge.net) to restrict automated search terms to strings not present in the English dictionary and thus reduce the false positive rate. This library includes 652,475 search terms which include all know English words and word versions (including British, American and Canadian spellings), as well as common abbreviations. Search terms included HGNC gene names, symbols and past symbols. Gene symbols with positive hits from this word library were only used as search strings in all capitals format, while gene names and past symbols present in SCOWL were excluded from manuscript searches. Once automated search results were compiled we manually confirmed the presence of each gene for which the automated search detected hits in 3 or more profiling papers, or within our key gene lists presented in Tables 1 and 2. Text search results, excluding the three studies from which we have drawn data, for our key gene lists were incorporated into Tables 1 and 2 (last column), as well as Table 4.

**Table 4:** Genes reported in at least 3 of the 16 esophageal expression profiling studies which compare squamous, BE and EAC tissue groups.

HGNC ID	SYMBOL*	Description*	Symbol#	allias1∧	allias2∧	Ref count	Profiling refs
6441	KRT4	keratin 4	KRT4	CYK4	CK4	8	[13], [15], [16], [17], [20], [30], [34], [35]
6415	KRT13	keratin 13	KRT13	MGC3781	CK13	7	[15], [16], [20], [21], [30], [34], [35]
6442	KRT5	keratin 5	KRT5	EBS2	KRT5A	7	[15], [18], [20], [21], [34], [35]
11755	TFF1	trefoil factor 1	TFF1	HPS2	D21S21	7	[18], [19], [20], [21], [34], [35], [36]
6446	KRT8	keratin 8	KRT8	CARD2	CYK8	6	[16], [17], [19], [30], [34], [35]
534	ANXA10	annexin A10	ANXA10	ANX14		5	[15], [18], [19], [35], [36]
1373	CA2	carbonic anhydrase II	CA2	CAII	CA-II	5	[15], [17], [18], [34], [36]
3555	FABP1	fatty acid binding protein 1, liver	FABP1	L-FABP		5	[14], [17], [19], [20], [35]
6443	KRT6A	keratin 6A	KRT6A	KRT6C	CK6C	5	[15], [17], [20], [34], [35]
6444	KRT6B	keratin 6B	KRT6B	KRTL1		5	[15], [16], [20], [34], [35]
10492	S100A2	S100 calcium binding protein A2	S100A2	S100L	CAN19	5	[15], [17], [18], [20], [36]
11757	TFF3	trefoil factor 3 (intestinal)	TFF3			5	[18], [19], [21], [34], [35]
3333	EMP1	epithelial membrane protein 1	EMP1	TMP	CL-20	4	[15], [17], [18], [36]
6187	IVL	involucrin				4	[15], [18], [20], [30]
6412	KRT1	keratin 1	KRT1	EHK1	KRT1A	4	[17], [20], [34], [35]
6416	KRT14	keratin 14	KRT14	EBS3	EBS4	4	[15], [20], [34], [35]
6427	KRT17	keratin 17	KRT17	PCHC1		4	[16], [20], [34], [35]
20412	KRT20	keratin 20	KRT20	CK20	K20	4	[14], [19], [34], [35]
6445	KRT7	keratin 7	KRT7	K2C7	CK7	4	[30], [34], [35], [36]
6565	LGALS4	lectin, galactoside-binding, soluble, 4	LGALS4	GAL4		4	[18], [19], [20], [35]
7512	MUC2	mucin 2, oligomeric mucus/gel-forming	MUC2			4	[14], [18], [30], [34]
7515	MUC5AC	mucin 5AC, oligomeric mucus/gel-forming	MUC5AC			4	[18], [21], [30], [34]
9273	PPL	periplakin				4	[13], [15], [17], [18]
10498	S100A8	S100 calcium binding protein A8	S100A8		CFAG	4	[15], [16], [20], [36]
10499	S100A9	S100 calcium binding protein A9	S100A9	CAGB	CFAG	4	[15], [18], [20], [36]
11244	SPINK1	serine peptidase inhibitor, Kazal type 1	SPINK1	Spink3	PCTT	4	[16], [18], [19], [21]
11263	SPRR2C	small proline-rich protein 2C (pseudogene)	SPRR2C			4	[15], [16], [17], [18]
11756	TFF2	trefoil factor 2	TFF2	SML1		4	[21], [34], [35], [36]
328	AGR2	anterior gradient homolog 2 (Xenopus laevis)	AGR2	XAG-2	HAG-2	3	[16], [17], [18]
533	ANXA1	annexin A1	ANXA1	ANX1	LPC1	3	[15], [17], [18]
546	ANXA8	annexin A8	ANXA8	ANX8		3	[15], [17], [18]
1805	CDX1	caudal type homeobox 1	CDX1			3	[20], [30], [34]
2481	CSTA	cystatin A (stefin A)		STF1		3	[16], [17], [18]
2500	CTGF	connective tissue growth factor	CTGF	IGFBP8	CCN2	3	[14], [16], [36]
2530	CTSE	cathepsin E	CTSE			3	[15], [18], [19]
3153	ECM1	extracellular matrix protein 1	ECM1			3	[17], [18], [36]
3690	FGFR3	fibroblast growth factor receptor 3	FGFR3	JTK4	CEK2	3	[15], [18], [21]
4164	GAST	gastrin				3	[13], [20], [36]
4174	GATA6	GATA binding protein 6	GATA6			3	[15], [18], [19]
5476	IGFBP7	insulin-like growth factor binding protein 7	IGFBP7	MAC25	IGFBP-7	3	[16], [17], [36]
6361	KLK13	kallikrein-related peptidase 13	KLK13	KLK-L4		3	[13], [15], [18]
6413	KRT10	keratin 10	KRT10	KPP	CK10	3	[18], [34], [35]
6421	KRT15	keratin 15	KRT15	K15	CK15	3	[15], [20], [34]
7511	MUC13	mucin 13, cell surface associated	MUC13	DRCC1		3	[13], [19], [34]
7517	MUC6	mucin 6, oligomeric mucus/gel-forming	MUC6			3	[18], [21], [30]
17190	OLFM4	olfactomedin 4	OLFM4	OlfD	GW112	3	[19], [20], [36]
8890	PGC	progastricsin (pepsinogen C)	PGC			3	[14], [20], [36]
9053	PLAUR	plasminogen activator, urokinase receptor	PLAUR	CD87		3	[15], [17], [30]
16	SERPINA3	serpin peptidase inhibitor, clade A, member 3	SERPINA3	AACT		3	[14], [18], [36]
10569	SERPINB3	serpin peptidase inhibitor, clade B, member 3	SERPINB3		SCCA1	3	[15], [17], [18]
9490	TMPRSS15	transmembrane protease, serine 15	TMPRSS15			3	[14], [20], [30]
17274	TRIM29	tripartite motif-containing 29	TRIM29	ATDC	FLJ36085	3	[15], [17], [18]
20657	TSPAN1	tetraspanin 1	TSPAN1	TSPAN-1	NET-1	3	[19], [21], [36]
11855	TSPAN8	tetraspanin 8	TSPAN8	TM4SF3	CO-029	3	[13], [15], [17]

*Capitalized HGNC gene symbols and descriptions were used as search parameters through each manuscript, excluding methods and supplementary material.

# For gene symbols not present as text strings within the English language, a separate case insensitive search was conducted of each manuscript.

∧ Additional searches were conducted using the previous gene symbols or abbreviations as listed in these “alias” columns.

### Support Vector Machine (SVM) analyses

By defining the overlap between the 4 cohorts (Table S1), and those present within at least 3 previous independent profiling studies (Table 4), we arrived at a list of 11 genes; CA2, ANXA10, CDX1, EMP1, IGFBP7, KRT1, KRT4, KRT20, LGALS4, TFF1 and TSPAN1. To estimate the utility of this list as a tissue type discriminator we applied a basic SVM LOOCV system using a first order polynomial kernel function and a diagonal scaling factor of one (GeneSpring GX version 7.3.1). Given that the two smaller cohorts (Gomes-41 and Hao-34) each contained data for only 4 of the 11 genes, they were excluded from the analysis. The two largest cohorts, SDH-54 and Greenawalt-102, contained transcripts of 11 and 10 of these genes respectively. From the resulting tables of expected and predicted tissue type assignments we calculated sensitivity and specificity using standard formulae [38].

## Results and Discussion

### Sample clustering

**Figure 2:**
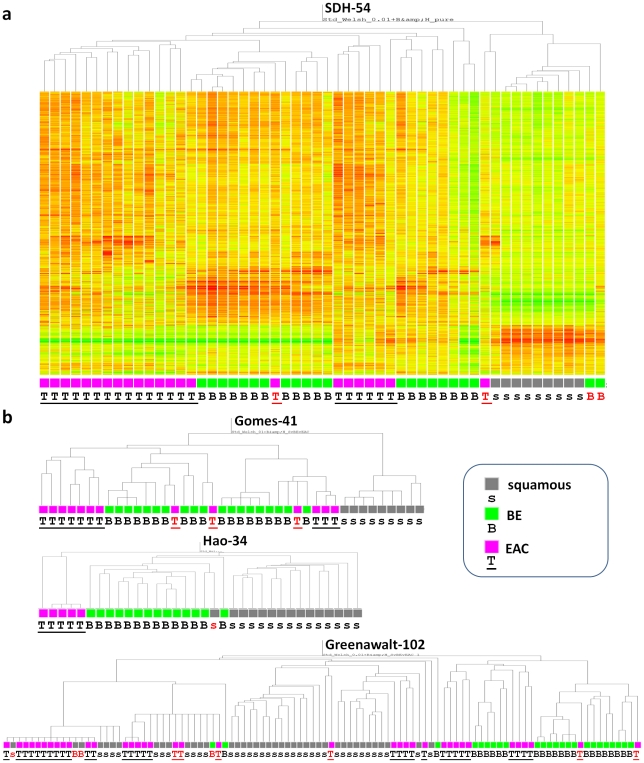
Supervised clustering in 4 cohorts to distinguish squamous, BE and EAC mRNA profiled samples. Cluster diagrams for a) the SDH-54 sample set introduced here and b) three previously published EAC cohorts for which raw data were publically available. Each dataset was clustered using the Genespring ‘Standard’ Algorithm. The gene lists used to cluster each cohort were generated using a Welsh ANOVA test to select genes that discriminate squamous, BE and EAC with a p value threshold <0.01. The Hao-34 sample set required a less stringent (p<0.05) threshold in order to generate genes. For each cohort squamous samples (s) are represented by grey boxes, BE (B) samples by green boxes and EAC (T) tumors by pink boxes. Samples highlighted in red indicate those that did not cluster as expected based on their expected pathology and are referred to in the text as ‘misclassified’.

The mRNA profiles of the squamous, BE or EAC biopsies from SDH-54 were clustered by the Genespring “Standard” clustering algorithm using those probes that significantly (p<0.01 after B&H false discovery adjustment) distinguished between the three tissue types. While this supervised clustering (Figure 2a) demonstrated relatively distinct squamous and columnar (BE+EAC) groups, there were some columnar samples (two BE and one EAC) that clustered with squamous tissues. The BE and EAC samples generally clustered as two distinct groups, with the exception of one EAC clustering within a BE group (Figure 2a). We expected to observe the three distinct sample types as distinct clusters, but analysis of data from 3 published studies [13], [16], [18] demonstrated similarly incomplete separation when the same analysis steps were applied (Figure 2b). Each dataset generally separated the squamous from the BE and EAC samples, but in all but one cohort there was incomplete separation between the BE and EAC specimens. These results are comparable to previously published cluster diagrams employing a variety of clustering methodologies to distinguish between esophageal tissues [13], [15], [16], [17], [18].

From Figure 2, the few samples that clustered unexpectedly in relation to their reported histology we henceforth refer to as ‘misclassified’. Across the 4 studies the samples ‘misclassified’ most often were EAC (11 out of 81; 13.6%, across the 4 studies), followed by BE (5/80; 6.3%). There were only 2 instances of squamous tissues clustering amongst BE or EAC groups (2/72; 2.8%). The ‘misclassification’ fraction varied between the different cohorts (Figure 2), with each research group having adopted a different strategy to attempt to enrich for tissue type within their samples, ranging from hand-dissected resections (1/34 or 2.9% ‘misclassification’) [13] to histology estimates of tissue content (10/102 or 9.8% ‘misclassification’) [18]. Not enough data were available to determine which was the better strategy, although none of these studies used micro-dissection (given the amount of mRNA required for whole-genome analysis) which is likely to be the superior approach in terms of controlling tissue purity [19], [39].

The higher rate of ‘misclassification’ amongst BE and EAC tissues could be explained in terms of contaminating epithelial tissue types, which would have had a concentration related impact on expression profiling. In the case of our SDH-54 dataset, we know that both of the EAC tumor samples that were ‘misclassified’ (Figure 2a) contained substantial copy number changes (data not shown) and around 60% tumor content [24], clearly distinguishing their DNA from that of either BE or normal squamous sample. These copy number data provide no explanation however, as to why one of these EACs would cluster amongst squamous samples and the other amongst BE on the basis of mRNA profiling.

Three of the four cohorts clustered in Figure 2 had a small number of EAC tissues that clustered with BE samples; one in SDH-54 (Figure 2a) two in Greenawalt-102 [18] and three in Gomes-41 [13] (Figure 2b). This was either the result of tumors with expression profiles similar to BE tissues, or those that contained a strong proportion of BE cells, in addition to the cancer. Looking at the available details from 4 of these EAC patients (sample 54043 from Table 1 of Nancarrow *et al* [24], as well as GH865, GH871 and HC03 from Table 1 of Gomes *et al* [13]; no additional data were available from the Greenawalt study) they ranged from tumor stage I to III, disease stage II to IV and included both moderate and poorly differentiated cancers. Thus it seems unlikely that these EAC samples represent a subset with a similar tumor profile to BE.

The SDH-54 cohort was the only one of the four studies to use BE tissue exclusively from participants with no histological evidence of either dysplasia or EAC. The two misclassified BE samples within the SDH-54 cohort clustered with the squamous samples, and neither showed evidence of copy number changes using genome-wide high-density SNP chip data (results not shown). Together these observations suggest that a mixture of tissue types within a biopsy is a key factor in sample misclassification.

We also conducted unsupervised clustering of the SDH-54 cohort, using the same clustering algorithm and all available uniquely binding probes [25]. The pattern was almost identical whether the clustering was supervised (Figure 1a) or unsupervised, but this was not the case for several of the other data sets (data not shown).

### Gene ontology

Using the procedure outlined in Figure 1, we applied a standard series of data enrichment steps to each cohort in order to derive discriminatory (S vs BE vs EAC) gene lists for each study. There were 8762 unique Entrez gene identifiers present in at least 3 of the studies; around one third of the human genome, assuming there are 20–25 K genes in total [40]. We combined the gene lists from each of the 4 independent studies, based on Entrez gene identifiers (see Figure 1), to create a single gene list (n = 2240) with sub-threshold Welsh test p values in at least 75% (3/3, 3/4 or 4/4) of tested studies to characterize the differences between squamous, BE and EAC tissues. To better define the involvement of key pathways, we applied fold change filters to this list (Figure 1) to distinguish the most active genes within the tissue group comparisons, and noted the direction of these changes. We selected those genes for which, in at least 3 studies, there was a fold change difference of 1.2 or greater for either the squamous to BE, or BE to EAC comparison (n = 851; Table S1) and subdivided this list based on the fold change direction for each comparison as shown in Figure 1. We used these sub-lists as the basis from which to investigate gene ontology changes, in order to identify the most important biological processes in the progression from squamous epithelium to BE and then EAC.

**Figure 3:**
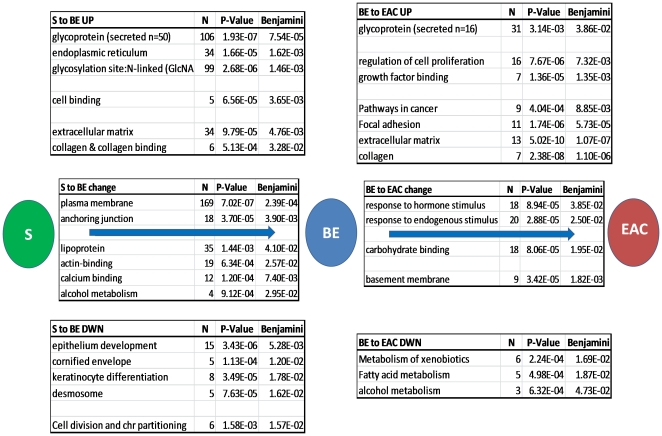
Gene ontology clusters significantly overrepresented in squamous to BE and BE to EAC comparisons across 4 cohorts. Genes with a >1.2 fold mean sample group comparisons for squamous (s) to BE and BE to EAC comparisons in at least 3 of the 4 cohorts were used, as presented in Figure 1. Statistically overrepresented ontology clusters were identified using DAVID, with all standard settings and a Benjamini false discovery adjusted p value threshold less than 0.05. Gene lists for squamous to BE and BE to EAC comparisons were subdivided on the basis of fold change direction (up or down regulated) and passed through DAVID separately. Gene clusters over-represented amongst genes over expressed in BE (left) and EAC (right) are presented on the top, while over-represented ontology groups amongst the under expressed genes in BE (left) and EAC (right) are tabulated on the bottom. Clusters in the middle of each comparison represent those over-represented on both the over and under expressed gene lists, indicating expression change.

The Entrez identifiers for each of these lists were then passed through the DAVID ontology website tool (using default settings), to catalog gene clusters overrepresented in each list. All ontology groups with Benjamini FDR adjusted scores less than 0.05 were considered. Given the frequent overlap between these networks of gene groups, we summarized the groupings in Figure 3 with the use of DAVID as a guide, and reported the most significant p value for each grouping. Any ontology groups that were present on both increasing and decreasing fold change lists were considered to be altered, as opposed to increased or decreased.

### Peak discriminating genes

When discussing ontology groups listed in Figure 3, we wanted to identify those genes with the strongest differences within our study as examples of each key gene group. By limiting the differentially expressed genes to those with the strongest group fold change differences, as shown in Figure 1, we have identified the most informative genes in the squamous to BE comparison (n = 76; Table 1 & 2) using a 3 fold cutoff and a >2 fold difference when BE was compared to EAC (n = 27; Table 3). Given the more pronounced tissue differences, as evident from the clustering experiments in Figure 2, there were more genes that consistently discriminated between BE and squamous tissues when compared to EAC and BE, hence the need for differential fold-change filters. It is of interest that a number of genes (ADH1C, ANXA10, CA2, HYAL1, KRT20, LIPF, MUC6, MYO1A, SULT1C2 and TFF1) appear on both the peak squamous to BE (Table 2) and BE to EAC (Table 3) comparison lists. In each case the expression level for these genes increased between squamous and BE, then decreased when BE was compared to EAC.

### Gene ontology

As the genes listed in Tables 1 and 2 provide the best indicators of particular ontology groups, their inclusions have been noted in the following summary of ontologies presented in Figure 3.

Epidermis development (CRABP2, BNC1 & EMP1), cornification (EVPL & PPL) and keratinocyte differentiation (AQP3) are all specific features of the stratified squamous epithelium. Figure 3 shows that genes from these ontology groups are overrepresented amongst mRNAs more highly expressed in normal esophageal squamous tissue, compared to BE, as previously reported [13], [15], [20], [30].

When BE and normal squamous expression profiles were compared, many more genes were up-regulated in BE, as were ontology groups related to the production of excreted glycoproteins. As seen in the upper left of Figure 3, we observed an increase in the mRNA levels of functional elements of the endoplasmic reticulum (ER) (ACSL5, AGR2, ANTXR2, ATP2A3, KDELR3, PDIA5 & PLOD3) and to a lesser extent Golgi apparatus (DPP4 & ITM2C), for which ontology was just below significance (data not shown), indicating increased glycosylation capacity within BE tissue, as well as a significant increase in secreted glycoproteins (ANTXR2, AZGP1P1, CFB, FMOD, HYAL1, LIPF, LUM, MUC3B & MUC6). Enlarged Golgi apparatus and prominent ER are required for increased glycoprotein biosynthesis, and electron microscopy studies have identified these features in BE [41], [42], providing physical support for the expression changes seen here. Perhaps the decreased expression of organelle size control genes, such as CDA and CRYAB (Table 1), reflect the need for these prominent structures in BE.

It has been proposed that, as with gastric epithelium, a key function of BE tissue is to protect against damage from luminal acid [43]. While there is not a designated ontology category for mucosal defense, our discriminating gene list (Table S1) includes several factors known to be involved in mucus barrier formation (MUC3B, MUC6 & TFF1), tight junction formation (CLDN11, CLDN15 & CLDN18), as well as carbonic anhydrases (CA2, CA9 & CA12) and solute carriers (SLC4A2 and SLC26A6) capable of generating and transporting HCO3- to protect against acidification [44] all of which are critical elements of a mucosal defence system [43]. Together these data support the hypothesis that a major role of BE tissue within the lower esophagus is to provide enhanced mucosal defense against the effects of erosive reflux [10], [21], [43], as evidenced by a much thicker mucosal barrier [45] and higher level of active ion transport [43], [46] compared to normal esophageal squamous epithelium.

Electron microscopy studies indicate that EAC tumors, and indeed advanced stage BE samples, appear to lose the well-developed Golgi apparatus and are not as adept at glycoprotein vesicle production [41]. While we note that the tumor tissue from patients with EAC showed evidence of reduced Golgi (RAB27A, AKR1B10) and ER (ENTPD5) activity compared with that of BE biopsies, neither of these ontology clusters were significantly over-represented amongst under-expressed EAC genes (data not shown). In fact there was an over-representation of secreted glycoproteins in EAC (Figure 3), including 7 of the 9 most over-expressed genes (COL1A2, COL4A1, COL4A2, COL5A2, COL6A3, IGFBP7 & PLAU) presented in Table 3, most of which (all but PLAU listed above) also showed altered expression levels in the squamous to BE comparisons (Table S1) and relate to the extracellular matrix (ECM). While it is true that ECM manipulation is an important aspect of tumor growth and invasion, it should be noted that there was very little support for these genes from amongst the other 13 expression profiling studies (Table 3).

We noted a reduced activity in gene ontology groups that relate to metabolic and xenobiotic activities (HPGD, LIPF, SULT1C2, ADH1B, ADH1C, ALDH3A1, AKR1C1, AKR1C2, AKR1B10) within EACs, as have other profiling studies [13], [15], [18], [19], [36]. These changes may signify dedifferentiation, a feature of cancer, and perhaps indicate that EAC cancer cells maybe more susceptible to the DNA damaging effects of smoking and reflux, although we could not find literature to support this.

Both MUC6 [47] and TFF1 [48] proteins are frequent constituents of adherent mucus and within BE tissue their decreased expression (in combination with other secreted mucins and trefoil factors) have previously been noted as an indication of early progression towards tumor development [20], [49], [50], [51], [52]. TFF1 is suspected of playing a direct role in mucus polymerization [48] and mucus viscosity [53], while CA2 is a key enzyme for reducing acidity through bicarbonate buffering [44]. Given that gastric acid can cause double-stranded DNA damage within exposed BE tissue [54], a breakdown in the mucosal defence system could contribute to the frequent chromosomal damage seen in EAC [24], [55], [56], [57]. More research is required in this regard.

Within the current 4 cohort study, we saw an over-representation of genes involved in growth factor binding (COL1A2, COL4A1, IGFBP7) and the regulation of cell proliferation (IGFBP7, NUP62, PLAU, STAT1), similar to several other expression profiling studies, although involving different subsets of genes [14], [15], [17], [39]. While cell cycle abnormalities are frequent events in cancer, Chao and coworkers demonstrated that they are not a feature of the progression from BE to EAC using a large, prospectively followed cohort of patients with BE [58]. It has been suggested that these and related observations indicate that abnormal cell cycle entry or exit may be responsible [59]. The p53 tumor suppressor protein is pivotally placed to control cell cycle entry/exit in response to DNA damage. Several studies indicate that the TP53 gene is frequently affected by mutation [60], [61], [62], [63] and copy number variation [24], [55], [64], [65], [66] within EAC, and that these changes are likely to increase protein stability, rather than mRNA levels, resulting in abnormal entry into the cell cycle without stopping for DNA repair (reviewed by Fitzgerald 2006 [59] and Reid 2010 [10]). It should also be noted that most of the above listed genes appear to have multiple functions, with many also active within the ECM. So while this result may be an indication of cell cycle/proliferation changes the listed genes are not well represented amongst other EAC profiling studies and the mode of their involvement is unclear.

### Comparison to other profiling studies

We have examined 13, independent array studies involving mRNA extracted from BE and EAC tissues to gauge how well represented our peak gene lists are within other papers (Table 1, 2 and 3). Over 45% (43/93) of the combined genes from our two peak lists were mentioned, either by name or official gene symbol, in at least one independent published BE-related array study. Of these, only 7 genes were described within 3 or more of the 13 independent profiling studies: EMP1, CA2, LGALS4, TFF1, TSPAN1, ANXA10 and KRT20 (Table 1, 2 and 3) plus another 6 genes (ANXA1, CDX1, CSTA, ECM1, KRT1 and KRT4) present within Table S1, suggesting their potential importance within this tissue progression. The most frequently implicated gene families across all 16 previous mRNA profiling studies were keratins, mucins, trefoils, annexins and S100 calcium binding proteins, described in 12, 7, 6 and 6 studies respectively (Table 4), all of which are represented within the peak lists of the current study, with the exception of S100 proteins, although S100A7 is present on the 851 gene list used for ontology testing (Table S1). Conversely, while collagen genes (COL1A2, COL4A1, COL4A2, COL5A2 & COL6A3) were well represented amongst the peak genes amplified in EACs within the current study (Table 2), this gene family was poorly represented amongst previous studies (Table 4). Several genes not in our peak list (FABP1, IVL, SPRR2C, S100A2, S100A8, S100A9, TFF2, TFF3, MUC2, MUC5AC, along with KRT 5, 6A, 6B, 7, 8, 13, 14 and 17) were also frequently discussed across the 16 previous profiling studies. Secreted mucins and trefoil factors are well represented amongst these frequently reported genes. When combined with the 4 study data analysis presented here, these results confirming the importance of mucosal defense related factors in the squamous-BE-EAC tissue progression, as originally reported by Ostrowski and co-workers [21].

### Support vector machine discriminator analyses

Using the above described 11 genes that overlap between our 4 cohort analysis, and the 13 independent profiling studies (CA2, ANXA10, CDX1, EMP1, IGFBP7, KRT1, KRT4, KRT20, LGALS4, TFF1 and TSPAN1) we have conducted SVM based analyses with the two largest cohorts (SDH-54 and Greenawalt-102). In each cohort LOOCV analysis resulted in high (>88%) sensitivity and specificity for discriminating BE from squamous, and while the specificity of determining EAC (cancer) from BE or squamous (non-cancer) was equally high, the sensitivity for each cohort was 73%. In each case this translates into an unacceptably high false negative rate with 5/23 and 9/37 EAC samples predicted to be BE for SDH-54 and Greenawalt-102 cohorts respectively. Thus for a clinically useful mRNA based discriminator additional genes are required to specifically distinguish these two tissue groups. An important aspect, and one that has not been taken into consideration in the current study, is to assess transcript levels across a broad range of BE samples with regards to cancer risk. Several reports have begun this task [51], [67] but large, dedicated cohorts are required.

This study represents the most powerful genome-wide EAC related expression study to date, combining data from 4 study cohorts, with a total of more than 70 samples in each tissue group. In order to combined these data we have employed the Welsh test with standard 0.01 (or 0.05 for the smaller Hao-34 cohort) B&H FDR-adjusted thresholds to each cohort before applying cross-platform informatics to align each set of chip features to Entrez gene IDs and tissue group fold-change filters to enable the compilation of unified gene lists for squamous/BE and BE/EAC comparisons (Figure 1). The fact that these samples were collected by 4 independent groups across three countries using very different criteria, and each profiling study was done with a different set of methodologies means the combined results are more universally applicable than any one study. However, the need to independently analyze samples from each study (due to the broad set of both technical and experimental differences) does weaken the study design and reduces the number of considered genes to less than half the number of currently active human Entrez IDs. Thus the gene lists presented should be considered as inclusive, rather than exclusive. Lastly, given the broad base of included BE material used for the combined analysis, particularly with respect to histological typing and patient origins (cancer versus non-cancer subjects), the importance of identified genes within the context of the BE-dysplasia progression needs to be the focus of subsequent, well-defined studies.

In summary we have described a new, whole-genome expression dataset focused on comparing esophageal squamous, BE and EAC tissue types. We have combined this dataset with the raw data from three previously published cohorts to allow comparable analyses of the same basic sample groups. We have used these four datasets to generate a list of genes differentially expressed between these three esophageal tissue states. We present ontology studies demonstrating that many of these discriminating genes are in biologically plausible pathways involved in the progression from normal squamous epithelium to BE, or BE to EAC. We further stratify this gene list to identify those with the strongest profiling capabilities, and that are broadly discussed in other EAC-related genome-wide mRNA expression papers, generating a list of 93 genes most likely to be useful as expression markers. These genes and pathways provide a basis for subsequent work, which will attempt to provide expression profiling discriminators specific for each tissue type. We believe the following design factors need to be considered in future studies which seek to develop gene-based tests to discriminate between the squamous, BE and EAC tissue types:

1. Address the primary confounding issues involved in sample preparation: i.e. cell type heterogeneity within samples, expression heterogeneity between samples
2. Address the secondary confounding issues involved in patient selection: i.e. disease stage, site of lesion, patient risk factor profiles (obesity, reflux and smoking). Overcoming these issues will require detailed patient eligibility criteria and considerably larger sample sizes, ideally using long-term, prospective cohort studies.
3. Use complementary data from converging technologies on the same tissue samples (mRNA expression, copy number, methylation and DNA sequencing data) to gain a deeper understanding of the key molecular events in esophageal carcinogenesis.

In time, we foresee molecular tests will be developed with sufficient specificity and sensitivity to augment, or perhaps replace, histological classification of tissues within the esophagus. To ensure translation into clinical practice, there will always be a need to reduce the complexity of high dimensional procedures and gene-sets, and so future studies must bear this in mind.

## Supporting Information

Table S1 **ANOVA and Fold-change Data for Discriminating Genes in Squamous to BE and BE to EAC Comparisons Across 4 Cohorts.** For inclusion genes must show a sub threshold (p<0.01 in SDH-54, Gomes-41 & Greenawalt-102, and p<0.05 in Hao-34, as described in the Methods) false discovery rate (FDR) adjusted p value, as well as a >1.2 fold mean sample group comparisons for squamous to BE and BE to EAC comparisons in at least 3 of the 4 cohorts. Columns A to C contain gene details, columns E to H contain Benjamini & Hochberg FDR adjusted Welsh p values for each study, columns J to M contain squamous to BE mean group fold change values for each study, columns O to R contain BE to EAC mean group fold change values for each study, columns T to W show the number of probes on each chip for a given gene for each study and column Y shows the fraction of studies that meet the p value + fold change requirements out of the total number of studies with representative probes for a given gene.
(XLSX)
